# Pathways linking AI dependence to psychological distress: the mediating role of self-esteem and the role of academic achievement

**DOI:** 10.3389/fnhum.2026.1877234

**Published:** 2026-08-11

**Authors:** Xu Liu, Zhenggui Gu, Lulu Shi

**Affiliations:** 1 School of Educational Sciences, Xinxiang University, Xinxiang, China; 2 Department of Human Development and Family Studies, Faculty of Human Ecology, Universiti Putra Malaysia, Serdang, Malaysia; 3 School of Music, Xinxiang University, Xinxiang, China; 4 School of Education, Weifang University of Science and Technology, Weifang, China

**Keywords:** academic achievement, AI dependence, psychological distress, self-esteem, time-lagged study

## Abstract

The growing integration of artificial intelligence (AI) into educational contexts has raised questions about whether reliance on AI tools is associated with students’ psychological well-being. This three-wave time-lagged survey examined temporally ordered associations between AI dependence (AD), self-esteem (SE), and psychological distress (PD), and evaluated academic achievement, indexed by cumulative grade point average (CGPA), as an additional predictor and potential moderator. Purposive sampling recruited undergraduate students from two universities in China. Of 947 baseline participants, 865 provided complete and valid data across all three waves. AD, SE, and PD were assessed using self-report measures, and CGPA was self-reported. PLS-SEM in SmartPLS 4.0 and supplementary analyses in SPSS 26.0 tested direct, indirect, and interaction associations. Greater AD was associated with higher PD and lower SE. SE was negatively associated with PD and showed a significant statistical indirect association between AD and PD. CGPA was positively associated with SE but was not directly associated with PD; its indirect association through SE was significant. The hypothesized moderating effects of CGPA were not supported. These findings identify SE as a plausible statistical pathway, but they do not establish a causal mechanism; restricted CGPA variability, gender imbalance, purposive sampling, self-report measurement, and incomplete cultural validation of the Chinese AI-dependence scale warrant cautious interpretation.

## 1 Introduction

The increasing adoption of AI technologies in educational settings is reshaping student engagement, information search, writing, problem-solving, and decision-making (Tarlochan, 2025; Mohamad Mozie et al., 2025; George and Wooden, 2023). Recent evidence suggests that a substantial proportion of university students regularly use AI-based tools in academic activities (Fošner, 2024; Dahri et al., 2024). These developments are not inherently harmful; AI can support learning when used adaptively. However, concern arises when students become dependent on AI for routine cognitive and academic tasks, because repeated externalization of thinking may reduce autonomous engagement, perceived competence, and confidence in one’s own academic judgment.

Psychological distress is also common among university populations, with anxiety and depressive symptoms frequently reported across different educational contexts (Al-Garni et al., 2025; Han S. S. et al., 2025; Haruna et al., 2025). The coexistence of increasing AI reliance and high levels of student distress does not itself establish a causal relation. Nevertheless, it creates a theoretically important question: whether AI dependence is linked to distress through students’ self-evaluative processes. The present study addresses this question by integrating Self-Determination Theory (SDT) and a self-system perspective.

### 1.1 AI dependence and psychological distress

AI dependence is conceptualized as excessive reliance on AI systems for cognitive task completion, reflecting a shift from internally regulated to externally supported information processing (Morales-Garcia et al., 2024). It is operationalized through self-reported reliance on AI in academic activities. Unlike general internet or smartphone overuse, AI dependence concerns reliance on external cognitive agents that may substitute for autonomous thinking, judgment, and competence development (Sparrow et al., 2011; Young, 1998; Kuss et al., 2014).

From an SDT perspective, well-being depends partly on the satisfaction of autonomy and competence needs (Ryan and Deci, 2024). Repeated reliance on AI for academic tasks may coincide with fewer opportunities for autonomous mastery and may therefore be associated with weaker perceived competence and greater vulnerability to stress. Emerging research on digital dependency, AI anxiety, and technology-related fatigue similarly reports associations between maladaptive technology reliance, reduced self-efficacy, and distress-related outcomes (Chen et al., 2025; Groenestein et al., 2024; Kim et al., 2025; Tian and Zhang, 2025; Zhang et al., 2026). These observations justify testing whether AI dependence is positively associated with subsequent psychological distress, while not presuming causation.

Longitudinal findings concerning related forms of digital dependence have been heterogeneous. Smartphone addiction has shown reciprocal or bidirectional associations with depressive symptoms in multiwave studies, including evidence that these associations may differ by gender (Shi et al., 2023; Zhang et al., 2023). In contrast, device-recorded screen time did not prospectively predict weekly mood in a 12-week study of university students (Bradley and Howard, 2023). These findings indicate that problematic dependence should be distinguished from duration of technology use and that reverse or reciprocal pathways remain plausible. However, longitudinal evidence specifically examining AI dependence, self-esteem, and psychological distress remains limited.

### 1.2 Self-esteem as an indirect statistical pathway

Self-esteem reflects a global evaluation of self-worth (Rosenberg, 1965) and is consistently associated with psychological adjustment. Lower self-esteem has been linked to greater anxiety, depression, and general distress (Al-Amer et al., 2025; Han Z. et al., 2025; Hao and Zhang, 2025). A combined SDT and self-system account suggests that AI dependence may be linked to distress partly because it is associated with weaker self-evaluation. If students attribute academic functioning to AI rather than to their own competence, reliance may correspond to diminished self-esteem, which in turn is associated with greater distress.

Empirical studies have shown that self-esteem can statistically account for indirect associations between behavioral, environmental, and mental-health variables (Li and Hao, 2025; Hao and Zhang, 2025). The present study therefore treats self-esteem as a theoretically grounded statistical pathway, not as proof of a causal mechanism. Because each construct was measured only once, the proposed ordering remains vulnerable to unmeasured confounding and reciprocal influence.

### 1.3 Academic achievement as a predictor and potential moderator

Academic achievement, indexed by CGPA, is an observed performance indicator rather than a latent psychological construct. It may nevertheless be relevant to the model because achievement can shape students’ perceived competence and self-worth. Higher achievement is generally associated with stronger academic self-efficacy and more favorable self-evaluations (Song et al., 2025; Martens and Pham, 2025; Zeb et al., 2025). Within SDT, successful academic performance may support perceived competence, which could in turn be linked to higher self-esteem and lower distress.

At the same time, the role of achievement may be conditional. High-achieving students might be less vulnerable to AI dependence because they have stronger academic skills and self-regulatory resources. Conversely, achievement may not protect students when academic pressure is high or when distress is driven by factors outside performance itself. Recent evidence further indicates that academic stress may be associated with increased AI dependence, burnout, and anxiety, suggesting that stress-related coping processes are directly relevant to the psychological risks of maladaptive AI reliance (Wang et al., 2026). Because academic stress and pressure were not measured in the present study, they are treated as important alternative explanations rather than as tested variables. This limitation is important when interpreting the nonsignificant moderation findings.

### 1.4 The present study

The present study tested a time-lagged model in which AI dependence was measured at Time 1, self-esteem and CGPA at Time 2, and psychological distress at Time 3. The separation of measurement occasions establishes the ordering of observations and reduces same-occasion response bias; however, it does not establish that changes in AI dependence preceded changes in self-esteem or distress because each construct was measured only once. Accordingly, the model is interpreted as a set of temporally ordered direct and indirect associations, not as a developmental trajectory or definitive causal process.

The study tested the following hypotheses:

*H1*: AI dependence is positively associated with psychological distress.

*H2*: AI dependence is negatively associated with self-esteem.

*H3*: Self-esteem is negatively associated with psychological distress.

*H4*: AI dependence is expected to show a significant statistical indirect association with psychological distress through self-esteem.

*H5*: Academic achievement is positively associated with self-esteem.

*H6*: Academic achievement is associated with psychological distress indirectly through self-esteem.

*H7*: Academic achievement moderates the associations between AI dependence and psychological distress, AI dependence and self-esteem, and self-esteem and psychological distress.

*H8*: Academic achievement moderates the indirect association between AI dependence and psychological distress through self-esteem.

**Figure 1:**
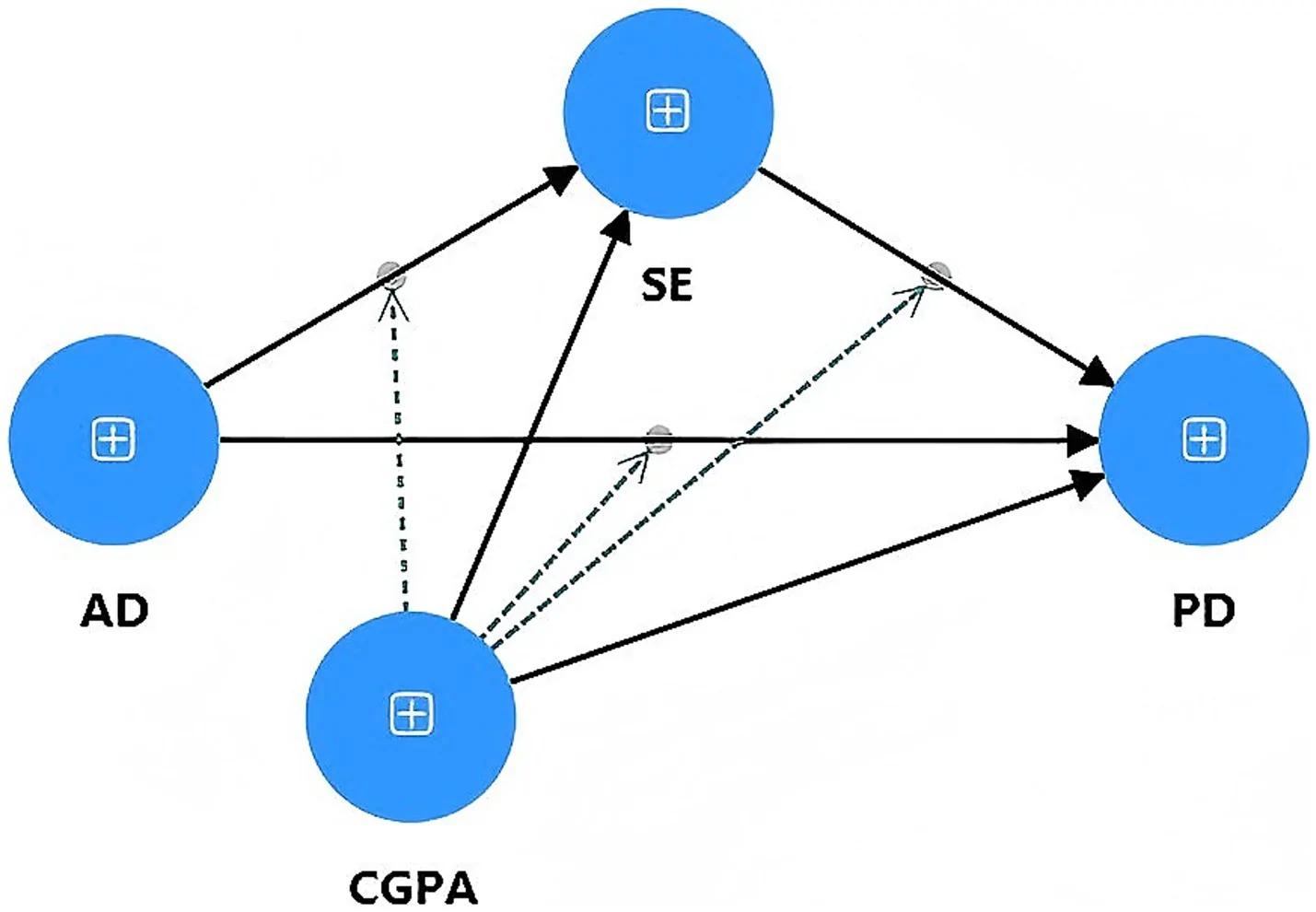
Hypothesized structural model linking AI dependence to psychological distress via self-esteem, with academic achievement as a predictor and potential moderator. Solid arrows represent hypothesized direct and indirect relationships; dashed arrows represent hypothesized moderation paths.

The hypothesized structural model is presented in Figure 1.

## 2 Method

### 2.1 Research design

This study used a three-wave time-lagged survey design. Variables were measured at different occasions separated by approximately 3 months: AI dependence and demographic information at Time 1 (November 2025), self-esteem and CGPA at Time 2 (February 2026), and psychological distress at Time 3 (May 2026). Separating measurement occasions reduced same-occasion response bias. Nevertheless, because each construct was measured once and no autoregressive or reciprocal paths could be estimated, this design cannot demonstrate within-person change, developmental trajectories, or causality.

### 2.2 Participants and sampling

Purposive sampling was used to recruit undergraduate students from two universities in China (Etikan et al., 2016). Participants were eligible if they were enrolled as undergraduates, were aged 18 years or older, had experience using digital learning tools, and were willing to complete all three waves. No minimum AI-use threshold was imposed. This non-probability strategy facilitated longitudinal recruitment but could over-represent students who were accessible, willing to participate, and already engaged with digital learning. Consequently, sampling limitations affect population generalizability even though they do not invalidate estimation of associations within the analytic sample.

At baseline, 947 students participated. After longitudinal matching and data quality screening, 865 participants aged 18–24 years provided complete and valid data across all waves and formed the analytic sample. The final sample exceeded recommended minimum sample size thresholds for PLS-SEM and provided adequate power for the tested structural paths (Hair et al., 2025).

### 2.3 Sample size estimation

*A priori* sample-size planning followed general SEM guidance and conservative recommendations for complex PLS-SEM models (Hair et al., 2025). The final sample of 865 exceeded the planned overall minimum. However, adequacy of the total sample does not guarantee equal precision in *post hoc* strata: the female group was substantially larger than the male group, and the relatively high CGPA distribution limited representation at the lower end of achievement. Any future confirmatory multi-group comparison should therefore use pre-specified strata, group-specific power analysis, and adequate recruitment within every stratum rather than relying only on the total *N*.

### 2.4 Instruments

#### 2.4.1 Psychological distress

Psychological distress was assessed using the K10 (Kessler et al., 2002), which measures the past-month frequency of anxiety and depressive symptoms on a 5-point scale (1 = never to 5 = always). Higher scores indicate greater distress. Internal reliability in this study was excellent (Cronbach’s alpha = 0.950).

#### 2.4.2 AI dependence

AI dependence was measured with the scale developed by Morales-Garcia et al. (2024), which assesses reliance on AI for learning, decision-making, and academic tasks. Items were rated on a five-point Likert scale, with higher scores indicating greater dependence. The original scale demonstrated a unidimensional structure, satisfactory reliability, construct validity, and gender invariance in university students. In the present sample, a Chinese version was administered and showed acceptable internal consistency (Cronbach’s alpha = 0.799). A formal independent back-translation procedure and a separate pilot or cognitive-interview study were not conducted before the main survey; these procedures are therefore not claimed retrospectively. This incomplete adaptation process creates possible semantic nonequivalence, culturally specific item interpretation, and construct-underrepresentation. Reliability within the present sample cannot by itself establish the psychometric validity of the Chinese adaptation.

#### 2.4.3 Self-esteem

The Rosenberg Self-Esteem Scale (Rosenberg, 1965) was used to assess global self-worth. Items were rated on a four-point scale, with higher scores indicating higher self-esteem. Internal consistency in this study was good (Cronbach’s alpha = 0.896).

#### 2.4.4 Academic achievement

Academic achievement was measured as self-reported cumulative grade point average (CGPA). CGPA was treated as a single observed variable rather than a latent construct.

### 2.5 Procedures and attrition

After ethics approval and authorization to use the copyrighted instruments, participants were recruited through WeChat, QQ, and university learning management systems. All participants completed the survey voluntarily after providing electronic informed consent. A unique identification code was used to match responses across waves while preserving anonymity.

Data collection occurred from November 2025 to May 2026. At Time 1, 947 students participated. A total of 865 students provided complete and valid data across all three waves, indicating an overall loss of 82 participants (8.7%) from baseline to the analytic sample. Cases were excluded if they could not be matched across waves or showed missing or inconsistent responses. Because wave-specific attrition counts were not available, attrition was reported from baseline to the final analytic sample.

### 2.6 Statistical analysis

SPSS 26.0 was used for data screening, descriptive statistics, correlations, and preliminary diagnostics. SmartPLS 4.0 (Ringle et al., 2024) was used to conduct PLS-SEM. PLS-SEM was selected because the study was prediction-oriented, included mediation and moderation paths, and used a model combining reflective latent variables with an observed academic performance indicator.

The measurement model was evaluated using indicator loadings, Cronbach’s alpha, composite reliability (rho_*c*), average variance extracted (AVE), and heterotrait-monotrait ratios (HTMT). The structural model was evaluated using standardized path coefficients, bootstrapped t values and confidence intervals, variance inflation factors (VIF), effect sizes (*f*^2^), *R*^2^, adjusted *R*^2^, and Stone-Geisser *Q*^2^ values. Bootstrapping tested direct, indirect, and interaction associations. A valid confirmatory comparison by gender or CGPA requires prior assessment of compositional measurement invariance (the MICOM procedure) and adequate group-specific power. Because verified MICOM and PLS-MGA outputs were unavailable, no subgroup path differences or measurement-invariance claims are reported. Accordingly, sample-distribution characteristics are discussed only as plausible explanations requiring future confirmatory testing, rather than as empirical subgroup findings.

## 3 Results

### 3.1 Preliminary findings

#### 3.1.1 Assessment of common method bias and data assumptions

Common method bias was assessed before hypothesis testing. The first factor accounted for 36.001% of the variance in Harman’s single-factor test, below the 50% criterion. Full-collinearity VIF values ranged from 1.010 to 1.117, below the conservative 3.3 threshold, suggesting that common method bias and multicollinearity were unlikely to drive the findings. Correlations among the main variables were small to moderate, and all structural VIF values were below conventional thresholds. Although these diagnostics supported the suitability of the data for PLS-SEM, future studies should report additional assumption checks, including skewness, kurtosis, residual patterns, and scatterplots, to provide a more comprehensive assessment of normality and linearity.

#### 3.1.2 Descriptive statistics and correlations

As shown in Table 1, the final sample (*N* = 865) included 611 females (70.6%) and 254 males (29.4%). Mean age was 19.56 years (SD = 1.15). The age distribution was recalculated from the reported frequencies: 459 participants were aged 18-19 years (53.1%), 222 were aged 20-21 years (25.7%), and 184 were aged 22 years or above (21.3%). The mean age at first smart-device acquisition was 13.55 years (SD = 2.79). The 2.4:1 female-to-male imbalance means that pooled estimates are more heavily informed by female participants and that male-specific estimates would be less precise.

**Table 1:** Sample characteristics.

Variable (*N* = 865)	Frequency (*n*)	Percentage (%)
Gender		
Female	611	70.6
Male	254	29.4
Age		
18 to 19	459	53.1
20 to 21	222	25.7
22 years and above	184	21.3
Mean	19.56	
SD	1.15	
Age at first smart-device acquisition		
5 to 10	121	14.0
11 to 17	651	75.3
18 to 20	93	10.8
Mean	13.55	
SD	2.79	

Percentages were calculated based on the final analytic sample (*N* = 865). Age-group percentages were derived from the reported frequencies.

Table 2 reports descriptive statistics and bivariate correlations. Academic achievement showed a relatively high mean (*M* = 3.33, SD = 0.43), indicating restricted representation of students at the lower end of CGPA. CGPA was positively correlated with AD (*r* = 0.101, *p* < 0.001) and SE (*r* = 0.195, *p* < 0.001), but was not significantly correlated with PD (*r* = −0.057, *p* > 0.05). AD was negatively correlated with SE (*r* = −0.082, *p* < 0.05) and positively correlated with PD (*r* = 0.312, *p* < 0.001). SE was negatively correlated with PD (*r* = −0.485, *p* < 0.001). The restricted CGPA range may attenuate interaction terms and is therefore a plausible sampling-based explanation for the nonsignificant moderation results, rather than evidence that achievement can never modify these associations.

**Table 2:** Descriptive statistics and correlations.

Variables	*M* ± SD	1	2	3
1. CGPA	3.33 ± 0.43			
2. AD	16.52 ± 3.15	0.101**		
3. SE	30.72 ± 4.67	0.195**	−0.082*	
4. PD	22.66 ± 7.76	−0.057	0.312**	−0.485**

AD, AI dependence; SE, self-esteem; PD, psychological distress; **p < 0.001; *p < 0.05.

### 3.2 Measurement model evaluation

The reflective measurement model demonstrated acceptable internal consistency, convergent validity, and discriminant validity. Outer loadings ranged from 0.549 to 0.883. Although one or more loadings were below the preferred 0.70 benchmark, the indicators were retained because construct-level reliability and AVE met acceptable thresholds and because item retention was theoretically justified.

Cronbach’s alpha values ranged from 0.799 to 0.950. Composite reliability (rho_*c*) values ranged from 0.815 to 0.953, and AVE values exceeded 0.50 for all reflective constructs (Table 3). HTMT values ranged from 0.165 to 0.509 (Table 4), all below the 0.90 criterion, supporting discriminant validity.

**Table 3:** Reflective measurement model.

Construct	Cronbach’s alpha	Composite reliability (rho_*c*)	AVE
AD	0.799	0.815	0.540
SE	0.896	0.914	0.513
PD	0.950	0.953	0.693

**Table 4:** Discriminant validity (HTMT).

Variables	1	2	3
1. AD			
2. SE	0.165		
3. PD	0.351	0.509	

HTMT values below 0.90 indicate acceptable discriminant validity.

These results support the internal psychometric performance of the Chinese version within the present sample; however, they do not constitute full evidence of cross-cultural validity or measurement invariance.

### 3.3 Structural model evaluation

#### 3.3.1 Direct and interaction effects

**Figure 2:**
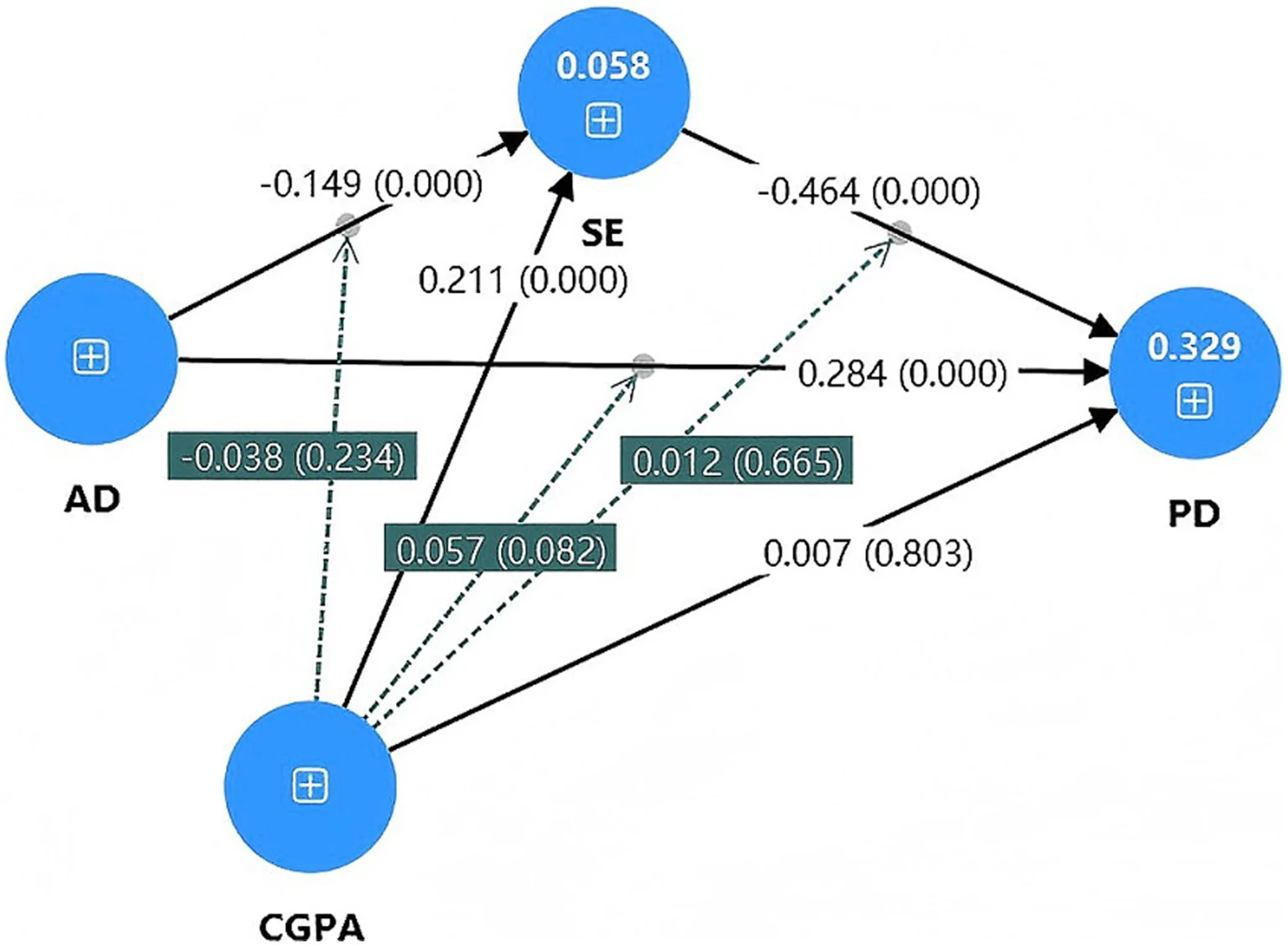
Structural model with standardized path coefficients. AI dependence was positively associated with psychological distress (beta = 0.284, *p* < 0.001) and negatively associated with self-esteem (beta = −0.149, *p* < 0.001). Self-esteem was negatively associated with psychological distress (beta = −0.464, *p* < 0.001). CGPA was positively associated with self-esteem (beta = 0.211, *p* < 0.001), but was not significantly associated with psychological distress (beta = 0.007, *p* = 0.803). The tested moderating effects were not significant.

As reported in Table 5 and Figure 2, AI dependence was positively associated with psychological distress (beta = 0.284, *t* = 9.297, *p* < 0.001, 95% CI [0.223, 0.344], *f*^2^ = 0.116), supporting H1. AI dependence was negatively associated with self-esteem (beta = −0.149, *t* = 4.059, *p* < 0.001, 95% CI [−0.223, −0.079], *f*^2^ = 0.024), supporting H2. Self-esteem was negatively associated with psychological distress (beta = −0.464, *t* = 16.625, *p* < 0.001, 95% CI [−0.520, −0.410], *f*^2^ = 0.302), supporting H3.

**Table 5:** Results of PLS-SEM analysis.

Predictor → Outcome	Beta	*t*	*p*	95% CI	VIF	*f* ^2^
Direct effects						
AD → PD	0.284	9.297	<0.001	[0.223, 0.344]	1.037	0.116
SE → PD	−0.464	16.625	<0.001	[−0.520, −0.410]	1.069	0.302
CGPA → PD	0.007	0.250	0.803	[−0.046, 0.064]	1.117	0.000
AD → SE	−0.149	4.059	<0.001	[−0.223, −0.079]	1.012	0.024
CGPA → SE	0.211	6.538	<0.001	[0.148, 0.274]	1.010	0.047
CGPA × AD → PD	0.057	1.737	0.082	[−0.008, 0.122]	1.029	0.005
CGPA × AD → SE	−0.038	1.190	0.234	[−0.101, 0.027]	1.002	0.002
CGPA × SE → PD	0.012	0.433	0.665	[−0.044, 0.068]	1.094	0.000
Indirect effects						
CGPA × AD → SE → PD	0.018	1.180	0.238	[−0.013, 0.047]		
AD → SE → PD	0.069	3.957	<0.001	[0.036, 0.105]		
CGPA → SE → PD	−0.098	5.875	<0.001	[−0.132, −0.067]		

CGPA was positively associated with self-esteem (beta = 0.211, *t* = 6.538, *p* < 0.001, 95% CI [0.148, 0.274], *f*^2^ = 0.047), supporting H5. CGPA was not directly associated with psychological distress (beta = 0.007, *t* = 0.250, *p* = 0.803, 95% CI [−0.046, 0.064], *f*^2^ = 0.000). The interaction effects CGPA × AD → PD, CGPA × AD → SE, and CGPA × SE → PD were not statistically significant (all *p* > 0.05), so H7 was not supported.

#### 3.3.2 Indirect effects

The statistical indirect effect of AD on PD through SE was significant (beta = 0.069, *t* = 3.957, *p* < 0.001, 95% CI [0.036, 0.105]), supporting H4. This finding indicates a significant indirect association but does not establish causal mediation. The statistical indirect effect of CGPA on PD through SE was also significant (beta = −0.098, *t* = 5.875, *p* < 0.001, 95% CI [−0.132, −0.067]), supporting H6. The moderated indirect effect involving CGPA × AD → SE → PD was not significant (beta = 0.018, *t* = 1.180, *p* = 0.238, 95% CI [−0.013, 0.047]); therefore, H8 was not supported.

#### 3.3.3 Explanatory power and predictive relevance

The model explained 33.2% of the variance in psychological distress (adjusted *R*^2^ = 0.329), indicating moderate explanatory power in behavioral research. The model explained 6.1% of the variance in self-esteem (adjusted *R*^2^ = 0.058), indicating limited explanatory power for this construct. Stone-Geisser *Q*^2^ values were above zero for psychological distress (*Q*^2^ = 0.121) and self-esteem (*Q*^2^ = 0.052), suggesting predictive relevance (see Table 6). However, the low *R*^2^ for self-esteem indicates that other factors, such as academic stress, social support, personality, and family or institutional context, likely contribute to self-esteem but were not included in the model.

**Table 6:** Structural model evaluation.

Endogenous construct	*R* ^2^	Adjusted *R*^2^	*Q* ^2^
PD	0.332	0.329	0.121
SE	0.061	0.058	0.052

## 4 Discussion

### 4.1 Overview of findings

This study examined associations linking AI dependence, self-esteem, academic achievement, and psychological distress within a time-lagged SDT and self-system framework. AI dependence was associated with higher psychological distress and lower self-esteem. Self-esteem was strongly and negatively associated with psychological distress and showed a significant statistical indirect effect in the association between AI dependence and distress. CGPA was positively associated with self-esteem and indirectly associated with lower distress through self-esteem, but it did not directly predict distress and did not moderate the tested pathways.

### 4.2 Integration with prior literature and theoretical contributions

The positive association between AI dependence and psychological distress is consistent with broader research on maladaptive technology reliance and psychological adjustment. Soraci et al. (2025), for example, reported structural associations among problematic social media and smartphone use, social phobia, and self-esteem. Although such technologies differ from generative AI, this literature provides a useful comparison because both involve potentially maladaptive reliance on digital tools. The present study extends this discussion to AI-supported academic work, but its contribution is preliminary because AI dependence remains a newer and less culturally validated construct.

Longitudinal evidence on related digital behaviors is more informative about directionality but remains heterogeneous. Four-wave evidence among Chinese college students found bidirectional within-person associations between smartphone addiction and depressive symptoms at later waves and an indirect pathway through loneliness (Shi et al., 2023). A two-wave study of freshmen likewise found reciprocal associations between smartphone addiction and depression, with the cross-lagged pattern evident among females but not males (Zhang et al., 2023). In contrast, a 12-week study using device-logged screen time found no week-to-week prospective association with mood, highlighting that duration of use is not equivalent to problematic dependence (Bradley and Howard, 2023). Together, these studies suggest that distress may precede, follow, or reciprocally reinforce problematic digital reliance depending on the construct, population, and measurement approach. The present time-lagged model adds evidence specific to AI dependence and self-esteem, but it cannot distinguish these competing directional accounts because the same constructs were not repeatedly measured.

The findings are also consistent with SDT. If students rely heavily on AI to complete academic tasks, they may have fewer opportunities to experience autonomous mastery, which may be associated with lower self-esteem and greater distress. The results do not demonstrate that AI dependence causes distress; rather, they support a temporally ordered association in which self-esteem is a plausible mediating pathway. This distinction is important given the observational design and modest explained variance for self-esteem.

A reverse pathway is also plausible: students experiencing anxiety, depressive symptoms, low confidence, or academic strain may turn to AI more frequently for reassurance, avoidance, decision support, or rapid task completion. Reciprocal reinforcement is also possible, whereby distress increases reliance and reliance subsequently coincides with weaker autonomous competence. The present design cannot adjudicate among these directions; repeated measurement of AD, SE, and PD and random-intercept cross-lagged modeling are needed.

### 4.3 Academic achievement and nonsignificant moderation

The positive association between CGPA and self-esteem suggests that academic achievement may matter most through students’ self-evaluative interpretation of performance. However, the relatively high CGPA mean and limited evidence about low-achieving students caution against generalizing this finding to all achievement levels.

None of the hypothesized moderating effects was supported. This does not establish that academic achievement is universally irrelevant as a boundary condition. The overall high CGPA and restricted dispersion reduce the variability needed to detect interaction effects, while the uneven gender composition may conceal heterogeneous patterns. The single self-reported CGPA indicator also introduces measurement error. Academic pressure, rather than attained grades, may be the more relevant moderator: recent evidence links academic stress with burnout and anxiety through AI dependence and diminished self-efficacy (Wang et al., 2026). Because stress was not measured and verified MICOM/PLS-MGA results were unavailable, subgroup explanations remain hypotheses for confirmatory, adequately powered research rather than findings of the present study.

The lack of a direct association between CGPA and psychological distress contrasts with research suggesting that academic performance and distress are often related. One explanation is that performance may not influence distress directly; rather, its psychological relevance depends on how students interpret achievement, whether they experience academic pressure, and whether performance supports or threatens self-worth. In the present model, self-esteem accounted for the CGPA-distress link statistically.

### 4.4 Limitations and future research

Several limitations should be considered. First, although the study used a three-wave time-lagged design, each construct was measured once. The findings show temporally ordered associations but cannot establish within-person change or causality. Psychological distress may increase reliance on AI, and reciprocal reinforcement is possible. Future panel studies should repeatedly measure AD, SE, and PD and use random-intercept cross-lagged models to separate stable between-person differences from within-person change.

Second, the study relied on self-report measures, including CGPA. Future research should incorporate official academic records, behavioral AI-use logs, and multi-informant data. Third, purposive recruitment from two Chinese universities, a predominantly female sample, and the high overall CGPA limit generalizability and may have reduced sensitivity to subgroup or moderation effects. A stronger follow-up design would recruit multiple universities across regions and institutional tiers; stratify by university, discipline, year, gender, and achievement band; randomly sample within strata; oversample underrepresented groups to meet group-specific power requirements; and apply sampling weights where appropriate.

Fourth, the Chinese adaptation of the AI-dependence scale was not supported by documented independent back-translation, a separate pilot/cognitive-interview study, or verified multi-group measurement-invariance output. Potential consequences include semantic drift, culture-specific response interpretation, differential item functioning, and biased structural coefficients. Future validation should use bilingual forward and back translation, expert reconciliation, cognitive interviews, an independent pilot sample, confirmatory factor analysis, convergent and criterion validity tests, and measurement invariance across gender, achievement, institution, and culture before substantive group comparisons. Fifth, wave-specific attrition and more detailed distributional diagnostics should be reported when available.

### 4.5 Practical implications for campus mental health

The findings support a tiered campus response focused on healthy AI use rather than prohibition. At the universal level, universities can embed AI literacy, independent problem-solving, source verification, self-monitoring, sleep protection, and clear boundaries for AI-assisted work into first-year and study-skills programs. Brief self-assessment tools can help students reflect on whether AI is augmenting or replacing autonomous learning. These recommendations concern psychosocial and cognitive well-being; the study did not measure neural outcomes and therefore does not demonstrate effects on brain structure or function.

At the selective level, counseling and academic-support services can jointly screen for problematic AI reliance alongside distress, loneliness, sleep problems, and academic strain, with confidential referral pathways for students reporting co-occurring difficulties. At the indicated level, trained practitioners may use motivational interviewing, cognitive-behavioral coping strategies, graded independent task practice, personalized AI-use plans, and peer or group support; students with clinically significant distress should receive appropriate professional assessment rather than AI-use advice alone. Implementation should be evaluated through uptake, acceptability, changes in autonomous study behavior, and mental-health outcomes. Because CGPA did not moderate the tested associations, support should remain available across achievement levels rather than assuming high-achieving students are protected.

The findings suggest that universities should promote balanced AI use that supports student autonomy and competence rather than discouraging AI use altogether. Instructional strategies may help students use AI as a learning aid while preserving independent reasoning, self-monitoring, and academic ownership. Because self-esteem was strongly associated with distress, student support programs should also attend to self-worth, competence beliefs, and healthy interpretations of achievement.

## 5 Conclusion

This three-wave time-lagged study found that AI dependence showed a positive direct association with later psychological distress and a positive statistical indirect association through lower self-esteem. Academic achievement was positively associated with self-esteem and showed a statistical indirect association with lower distress through self-esteem, but it was not directly associated with distress and did not moderate the tested pathways. These findings identify self-esteem as a plausible statistical pathway rather than a definitive causal mechanism. The results should be interpreted cautiously because the study was observational, relied on self-report data, and explained only a modest proportion of variance in self-esteem. Future work should examine reciprocal processes, academic stress, objective AI-use patterns, and broader contextual predictors of student mental health.

## Data Availability

The original contributions presented in the study are included in the article/supplementary material, further inquiries can be directed to the corresponding author.
